# Health status, respiratory symptom and dyspnea trajectories in subjects with chronic obstructive pulmonary disease: a seven-year observation in clinical practice

**DOI:** 10.1186/s41687-025-00923-z

**Published:** 2025-07-11

**Authors:** Koichi Nishimura, Masaaki Kusunose, Ayumi Shibayama, Kazuhito Nakayasu

**Affiliations:** 1 National Center for Geriatrics and Gerontology, 7-430 Morioka-cho, Obu, Aichi, 474-8511 Japan; 2 Clinic Nishimura, 4 − 3. Kohigashi, Kuricho, Ayabe, Kyoto, 623-0222 Japan; 3 Department of Respiratory Medicine, National Center for Geriatrics and Gerontology, 7-430 Morioka-cho, Obu, Aichi, 474-8511 Japan; 4 Department of Nursing, National Center for Geriatrics and Gerontology, 7-430 Morioka-cho, Obu, Aichi, 474-8511 Japan; 5 Data Research Section, Kondo Inc., 2-2-20, Kyomachibori, Nishi-ku, Osaka-shi, Osaka, 550-0003 Japan

**Keywords:** Chronic obstructive pulmonary disease, Health status, Dyspnea, Patient-reported outcome measures, Quality of life

## Abstract

**Background:**

Chronic obstructive pulmonary disease (COPD) is characterized by progressive airflow limitation, often associated with declining health status. It is widely believed that the burden of the disease increases over time, leading to continuous suffering in the patient. Understanding the long-term course of patient-reported outcomes (PROs) and the variability in disease progression is crucial for effective management. The purpose of this research was to investigate the long-term trajectories of health status, respiratory symptoms, and dyspnea in COPD patients over a seven-year period and to identify factors associated with different progression patterns.

**Methodology:**

This longitudinal study followed 70 COPD patients for seven years, with evaluations every six months. Participants underwent pulmonary function tests and completed four PRO measures: St. George’s Respiratory Questionnaire (SGRQ), COPD Assessment Test (CAT), Evaluating Respiratory Symptoms in COPD (E-RS), and Dyspnoea-12 (D-12). Annual changes were estimated using linear mixed models and linear regression analysis. The patients were categorized into quartiles based on the rate of decline in forced expiratory volume in one second (FEV_1_) and changes in PROs.

**Results:**

The group showed a significant deterioration in the FEV_1_ and PRO measures. FEV_1_ declined by 25 milliliters annually, while SGRQ Total and CAT scores worsened by 1.4 and 0.6 units per year, respectively. However, substantial variability was observed between individuals. The SGRQ Total score worsened significantly after 1.0 year in the 4th quartile, while the 1st quartile showed improvements at 2.0, 2.5, 3.0, 4.0, 6.0 and 6.5 years. Similarly, while the CAT, E-RS Total and D-12 Total scores deteriorated in the fourth quartile, they remained stable or improved in the first quartile.

**Conclusions:**

The progression of COPD varies widely among individuals. Although some patients experience significant declines, others remain stable or even improve for seven years. These findings challenge the belief that COPD inevitably leads to a constant increase in the burden of disease.

**Supplementary Information:**

The online version contains supplementary material available at 10.1186/s41687-025-00923-z.

## Background

Since the initial recognition of chronic obstructive pulmonary disease (COPD) as a distinct disease, it has been widely acknowledged that airflow limitation is one of its crucial characteristics, gradually deteriorating over time, with forced expiratory volume in one second (FEV_1_) decreasing each year [1, 2]. Along with the progress of airflow limitation, the clinical signs of COPD have been considered to progress in a similar fashion, suggesting a cycle of deterioration that may be associated with a poor outlook after a COPD diagnosis [3, 4]. 

Other measures of disease status in COPD have been introduced into clinical practice since the end of the 20th century. These measures aim to objectively describe how individual patients are affected by the disease. Patient-reported outcomes (PRO) such as health-related quality of life and health status are now being used to assess the impact of COPD in patients [5–11]. Several studies have found a weak correlation between physiological measures such as FEV_1_ and PRO measures [12, 13]. 

The decline in health status of COPD patients was initially investigated in the Inhaled Steroids in Obstructive Lung Disease (ISOLDE) trial [14]. Participants were asked to complete the St. George’s Respiratory Questionnaire (SGRQ) every six months for a period of three years. The findings revealed that the SGRQ Total score deteriorated faster with a placebo (3.2 units/year) than with inhaled corticosteroids (2.0 units/year) [4, 14]. 

The SGRQ is a widely used tool for measuring COPD-specific health status [7], but simpler PRO tools with fewer items are now preferred. Three simple PRO tools have been developed: the Evaluating Respiratory Symptoms in COPD (E-RS) for assessing respiratory symptoms [15, 16], the Dyspnoea-12 (D-12) for assessing breathlessness [17–19], and the COPD Assessment Test (CAT) for assessing COPD-specific health status [20–23]. In this cohort study conducted at our outpatient clinic, the authors administered the E-RS, D-12, CAT, and SGRQ over a number of years. The aim was to investigate the longitudinal patterns of these PRO measures over a seven-year period, reflecting the clinical course of COPD.

## Methods

### Study design and patients

This longitudinal cohort study was conducted at the National Center for Geriatrics and Gerontology (NCGG) in Japan from 2013 to 2023. Patients were recruited from the outpatient respiratory clinic based on the following inclusion criteria: age ≥ 50 years, a smoking history of ≥ 10 pack-years, and a post-bronchodilator FEV_1_/forced vital capacity (FVC) ratio below 0.7 [24, 25]. At baseline they were required to have been under our outpatient clinic’s care for at least six months to prevent confounding factors from new treatments. Exclusion criteria included a history of asthma, unresolved comorbidities, and COPD exacerbations defined as a worsening of respiratory symptoms requiring treatment with systemic corticosteroids or antibiotics, or both [26] in the three months prior to each reassessment. The study protocol was approved by the Ethics Committee of the National Center for Geriatrics and Gerontology (No. 1138–3) (updated on 12 July 2020), adhered to the Declaration of Helsinki and written informed consent was obtained from all participants.

Patients who were eligible for the study were enrolled without restrictions on COPD treatment or management from September 2013, with plans to follow them for a seven-year period. The study entry was completed in March 2016, with the final assessment scheduled for March 2023. Patients were excluded from subsequent reassessments if they had experienced an acute exacerbation within the preceding three months. In such cases, the reassessment was postponed ensuring the exclusion criteria were not violated. Assessments could also be postponed for personal reasons of the participant, with the description stating that the assessment was to be conducted as close as possible to the scheduled date. As the study sought to assess the course and speed of decline in lung function measurements and PRO measures, statistical methods required the inclusion of at least three measurements for analysis.

### Study assessments

Participants underwent pulmonary function tests at baseline and at each subsequent visit, with the PRO questionnaires being administered. They were instructed to come in at least 12 h after stopping bronchodilator use. Spirometry was performed for at least 60 min after inhaling long-acting bronchodilators, supervised by a physician. Residual volume (RV) was measured using the closed-circuit helium method and predicted lung function values were calculated according to the guidelines of the Japanese Respiratory Society [27]. 

The study used validated Japanese versions of PRO measures [12, 25, 28, 29]. The PROs were administered before pulmonary function tests. These tools were self-administered in a paper questionnaire format under site supervision. Disease-specific health status was evaluated using the SGRQ and CAT. The SGRQ comprises 50 items categorized into Symptoms, Activity, and Impact components, with a Total score indicating overall health status [7]. Higher SGRQ scores reflect greater impairment. CAT scores range from 0 to 40, with 0 indicating no impairment [20–23]. Dyspnea severity was assessed using the D-12, which includes twelve items (seven physical and five affective) graded on a scale of 0 to 3, yielding a total score ranging from 0 to 36 [17–19]. Higher scores indicate more severe breathlessness. The E-RS total score, ranging from 0 to 40, reflects the severity of respiratory symptoms, with subscales for breathlessness (RS-Breathlessness), cough and sputum (RS-Cough and Sputum), and chest-related symptoms (RS-Chest Symptoms) [15, 16]. While the E-RS is typically electronic, the Japanese version was paper-based because of device unavailability [30, 31]. 

### Statistical analysis

The backgrounds were compared using the Kruskal-Wallis test or Fisher’s exact test. The annual changes (slopes) in FEV_1_ and PRO measures for the entire analysis population were estimated using a linear mixed model, with each metric as the dependent variable, time (years) as a fixed effect, and subjects as a random effect. No covariate adjustments were made. To estimate the means at each measurement point, we used a linear mixed model with time point as a fixed effect and subjects as a random effect, obtaining the estimated means and their 95% confidence intervals (CI). The Type III test of fixed effects was used to determine whether the values of each metric remained constant over time (measurement points). Pairwise comparison tests were also conducted to examine whether there were significant differences from the baseline values. The Bonferroni method was used to adjust for multiplicity of p-values. Annual changes in each metric for individual cases were determined by linear regression analysis. Annual changes were divided into quartiles, that is, we divided the data set into four equal parts, each containing 25% of the data, and the estimated means for subgroups were calculated again using a linear mixed model. The relationship between patient backgrounds and annual changes was analyzed using linear regression analysis, estimating the annual change per unit change. Spearman rank correlation tests were performed to examine relationships among other parameters. Summary statistics are expressed as mean ± standard error or estimated mean and their 95% CI. All analyses were conducted using SPSS Statistics, version 28.0 (IBM Corp.) and p-values less than 0.05 were considered to be statistically significant.

## Results

### Subject characteristics

A total of 70 participants (64 men) were included in the present study. At the seven-year time point, 37 participants were still alive, 21 had passed away, and information was not available for 12 participants. Only four participants had completed all 15 semi-annual assessments over the seven-year period, while 14 participants had completed 14 assessments, the highest number. Participants were divided into subgroups according to the number of assessments they had completed, but no significant differences were found in their values (Table S1 in the Additional File). The mean age and FEV_1_ at baseline were 74.2 years and 1.71 L (68.7% predicted), respectively. According to the Global Initiative for Chronic Obstructive Lung Disease (GOLD), a non-profit organization established by the World Health Organization and the US National Heart, Lung, and Blood Institute in 1997 to improve care for COPD, classification of airflow limitation [32], 24 participants (34.3%) were classified as GOLD 1, 32 (45.7%) as GOLD 2, 9 (12.9%) as GOLD 3, and 5 (7.1%) as GOLD 4.

### Seven-year progress of each indicator for the group as a whole

**Fig. 1:**
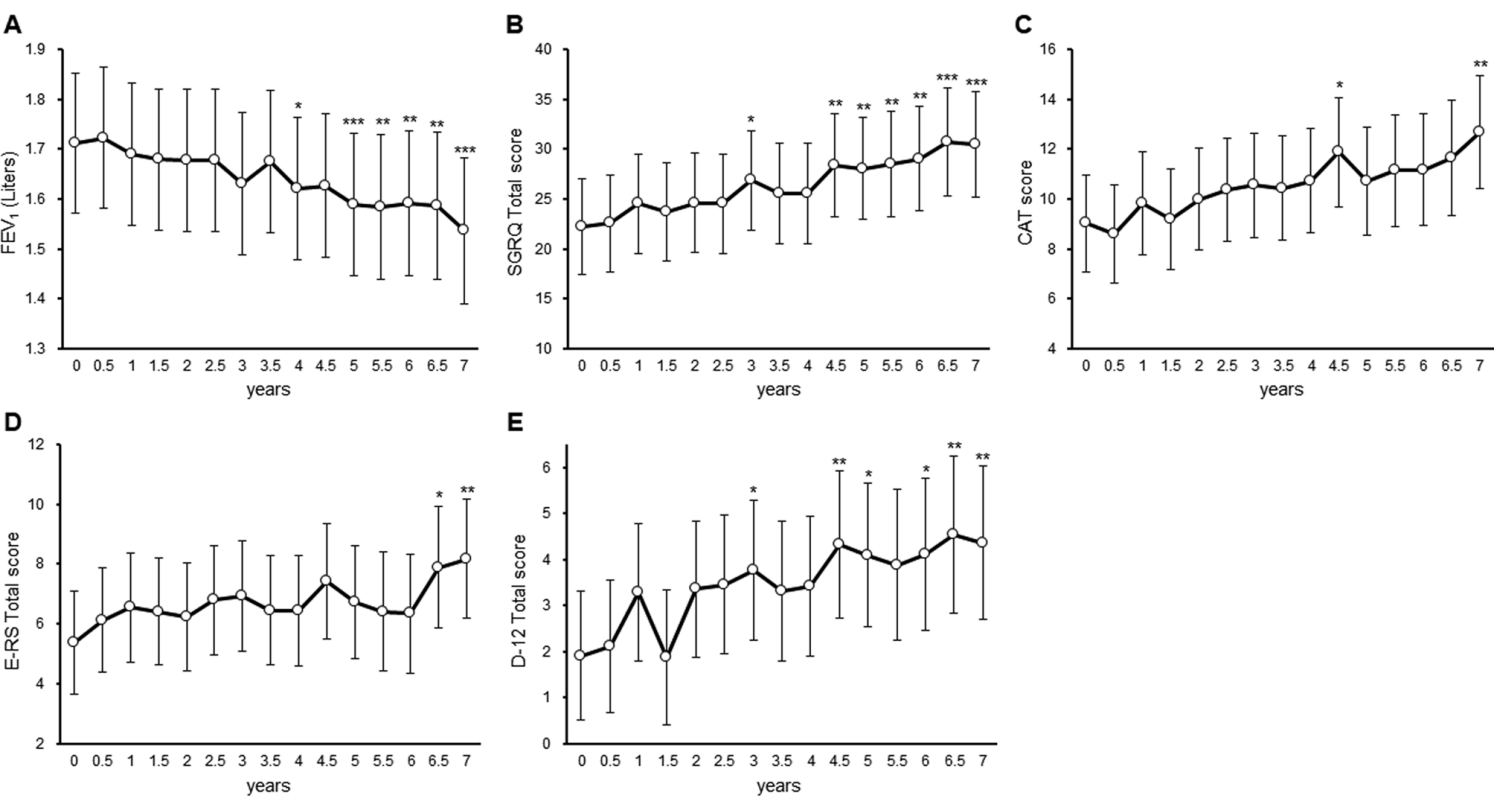
Changes in measured values over seven years, as calculated using a linear mixed model. The estimated mean and its 95% confidence interval of each indicator by a linear mixed model for each measurement point every 6 months during a 7-year period. **A**: forced expiratory volume in 1 s (FEV_1_) (Liters), **B**: St. George’s Respiratory Questionnaire (SGRQ) Total score, **C**: COPD Assessment Test (CAT) score, **D**: Evaluating Respiratory Symptoms in COPD (E-RS) Total score, **E**: Dyspnoea-12 (D-12) Total score. ***: *p* < 0.001, **: *p* < 0.01, *: *p* < 0.05 in comparison with baseline

Over the seven-year period, the group as a whole showed significant declines in several indicators (Table 1). On average, FEV_1_ decreased by 25 milliliters per year, while SGRQ Total and CAT scores deteriorated by 1.4 and 0.6 units per year, respectively. Although most PRO measures showed significant declines, the annual decreases in SGRQ Symptoms, E-RS Cough and Sputum, and E-RS Chest Symptoms scores did not reach statistical significance. The data analysis was conducted using a linear mixed model to account for within-subject variability in the study population (Fig. 1 and Table S2 in the Additional File). The estimated means and 95% CI for each measurement point were determined, showing a decrease in FEV_1_ over time, with a significant difference observed at four years compared to baseline. The SGRQ Total score increased slowly over the seven years, with a significant difference seen at three years. Similar trends were observed for the CAT score, with a significant difference at 4.5 years compared to baseline. The E-RS and D-12 Total scores also showed significant differences at 6.5 years and three years, respectively. Type III tests were statistically significant for FEV_1_, SGRQ Total, CAT, and D-12 Total scores, but not for the E-RS Total score.

**Table 1:** Patient characteristics at baseline and annual change estimated by the linear mixed model

	Units	Baseline data†			Annual change (/year) §			*p*-value	
FEV_1_	Liters	1.71	±	0.07	-0.025	±	0.006	< 0.001	
TLC	Liters	5.98	±	0.23	-0.115	±	0.026	< 0.001	
DLco	mL/min/mmHg	12.90	±	0.65	-0.732	±	0.096	< 0.001	
SGRQ Total Score	(0-100)	22.2	±	2.0	1.4	±	0.4	< 0.001	
SGRQ Symptoms	(0-100)	37.7	±	2.3	0.7	±	0.4	0.107	
SGRQ Activity	(0-100)	30.2	±	2.9	2.0	±	0.5	< 0.001	
SGRQ Impact	(0-100)	13.1	±	1.7	1.2	±	0.4	0.002	
CAT Score	0–40	9.0	±	0.8	0.6	±	0.2	0.002	
E-RS Total Score	(0–40)	5.4	±	0.7	0.3	±	0.1	0.038	
RS-Breathlessness	(0–17)	2.5	±	0.4	0.3	±	0.1	0.007	
RS-Cough & Sputum	(0–11)	1.9	±	0.2	0.0	±	0.0	0.346	
RS-Chest Symptoms	(0–12)	1.0	±	0.2	0.1	±	0.0	0.071	
D-12 Total Score	(0–36)	1.9	±	0.4	0.5	±	0.2	0.004	
D-12 Physical Score	(0–21)	1.6	±	0.3	0.2	±	0.1	0.010	
D-12 Affective Score	(0–15)	0.3	±	0.1	0.2	±	0.1	0.004	

Plus–minus values are means ±SE. †mean ±SE at baseline, §Annual change (mean ±SE) estimated by the linear mixed model, SGRQ, the St. George’s Respiratory Questionnaire; CAT, the COPD Assessment Test; E-RS, the Evaluating Respiratory Symptoms in COPD; D-12, Dyspnoea-12. The numbers in parentheses denote possible score range

### Annual rates of change in each indicator over a seven-year period

**Fig. 2:**
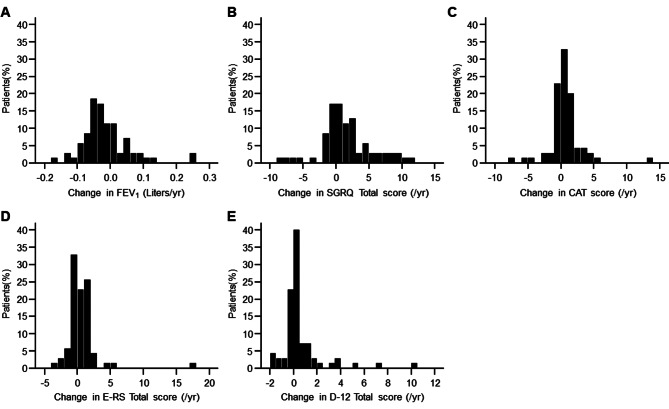
Frequency distribution of annual rates of change determined by linear regression analysis of individual cases. **A**: forced expiratory volume in 1 s (FEV_1_) (Liters), **B**: St. George’s Respiratory Questionnaire (SGRQ) Total score, **C**: COPD Assessment Test (CAT) score, **D**: Evaluating Respiratory Symptoms in COPD (E-RS) Total score, **E**: Dyspnoea-12 (D-12) Total score

Linear regression analysis of each indicator was performed for each case and the coefficient (slope) of the regression equation was tabulated as the annual change for each individual case. Frequency distributions of annual rates of change in FEV_1_, SGRQ Total, CAT, E-RS Total, and D-12 Total scores are shown in Fig. 2A to E. The Shapiro-Wilk test resulted in *p* < 0.001 for all indicators except for the SGRQ Total score, which was *p* = 0.005. As shown in Fig. 1 in the overall group, there was a general trend of slow deterioration in each indicator. However, some indicators worsened, some remained stable, and some even showed improvement, indicating that there was considerable variation between cases.

### Relationship with annual rates of change in each indicator

The relationships between patient characteristics or various clinical parameters at baseline and annual rates of change in FEV_1_, as well as the SGRQ Total, CAT, E-RS Total, and D-12 Total scores determined by linear regression analysis of individual cases, were examined (see Table 2 and S3 in the Additional File). However, no statistically significant predictors of the rate of these changes were found. Some correlation coefficients, for example, between baseline diffusion capacity of the lung (DLco) and the annual rate of change in FEV_1_ and the SGRQ Total score, were statistically significant at *p* < 0.05, but the correlations were quite weak. There was no correlation between the annual rates of change in FEV_1_ and the scores of the four PRO measures. However, there was a statistically significant correlation between the annual rates of change in any two of the scores of the four PRO measures (Spearman’s rho = 0.513 to 0.676).

**Table 2:** Correlation coefficients between annual rate of change and various clinical parameters

	Annual Rate of Change in FEV_1_ (ml/yr)		Annual Rate of Change in SGRQ Total Score		Annual Rate of Change in CAT Score		Annual Rate of Change in E-RS Total Score		Annual Rate of Change in D-12 Total Score	
	Spearman’s rho (ρ)	*p*-value	Spearman’s rho (ρ)	*p*-value	Spearman’s rho (ρ)	*p*-value	Spearman’s rho (ρ)	*p*-value	Spearman’s rho (ρ)	*p*-value
At baseline										
FEV_1_ (mL)	0.061	0.616	-0.311	0.009*	-0.202	0.094	-0.275	0.021*	-0.162	0.179
TLC (Liters)	-0.021	0.865	0.002	0.989	0.116	0.337	0.194	0.107	0.086	0.480
DLco (mL/min/mmHg)	0.275	0.021*	-0.237	0.048*	-0.168	0.165	-0.155	0.200	-0.216	0.072
Blood eosinophil count (%)	0.200	0.096	-0.075	0.537	-0.081	0.504	-0.074	0.544	0.040	0.739
Blood eosinophil count (cells/mm^3^)	0.188	0.119	-0.044	0.720	-0.058	0.632	-0.039	0.748	0.088	0.467
Erythrocyte sedimentation rate (mm/hour)	-0.031	0.796	-0.170	0.159	-0.224	0.063	-0.304	0.011*	-0.140	0.247
C-reactive protein (µg/mL)	0.201	0.099	-0.062	0.616	-0.095	0.443	-0.149	0.227	-0.012	0.922
Surfactant protein D (ng/mL)	0.106	0.392	0.029	0.816	-0.123	0.321	-0.053	0.673	0.099	0.426
SGRQ Total Score	0.046	0.707	0.132	0.274	0.134	0.269	0.085	0.483	0.319	0.007*
CAT Score	0.098	0.419	0.291	0.015*	0.046	0.704	0.202	0.093	0.332	0.005*
E-RS Total Score	0.031	0.798	0.173	0.152	0.224	0.062	-0.001	0.993	0.225	0.061
D-12 Total Score	0.068	0.574	0.175	0.146	0.205	0.089	0.082	0.501	0.156	0.196
Annual rate of change										
TLC	0.268	0.025*	0.077	0.526	-0.017	0.889	0.072	0.555	0.155	0.199
DLco	-0.129	0.288	-0.111	0.362	-0.104	0.390	-0.050	0.679	0.037	0.762
SGRQ Total Score	-0.074	0.541	-	-	-	-	-	-	-	
CAT Score	-0.128	0.291	0.610	< 0.001*	-	-	-	-	-	
E-RS Total Score	-0.025	0.838	0.676	< 0.001*	0.664	< 0.001*	-	-	-	
D-12 Total Score	-0.149	0.217	0.609	< 0.001*	0.513	< 0.001*	0.517	< 0.001*	-	

* Statistically significant relationships (*p* < 0.05). SGRQ, the St. George’s Respiratory Questionnaire; CAT, the COPD Assessment Test; E-RS, the Evaluating Respiratory Symptoms in COPD; D-12, Dyspnoea-12

### Seven-year trajectories in each indicator divided by quartiles

**Fig. 3:**
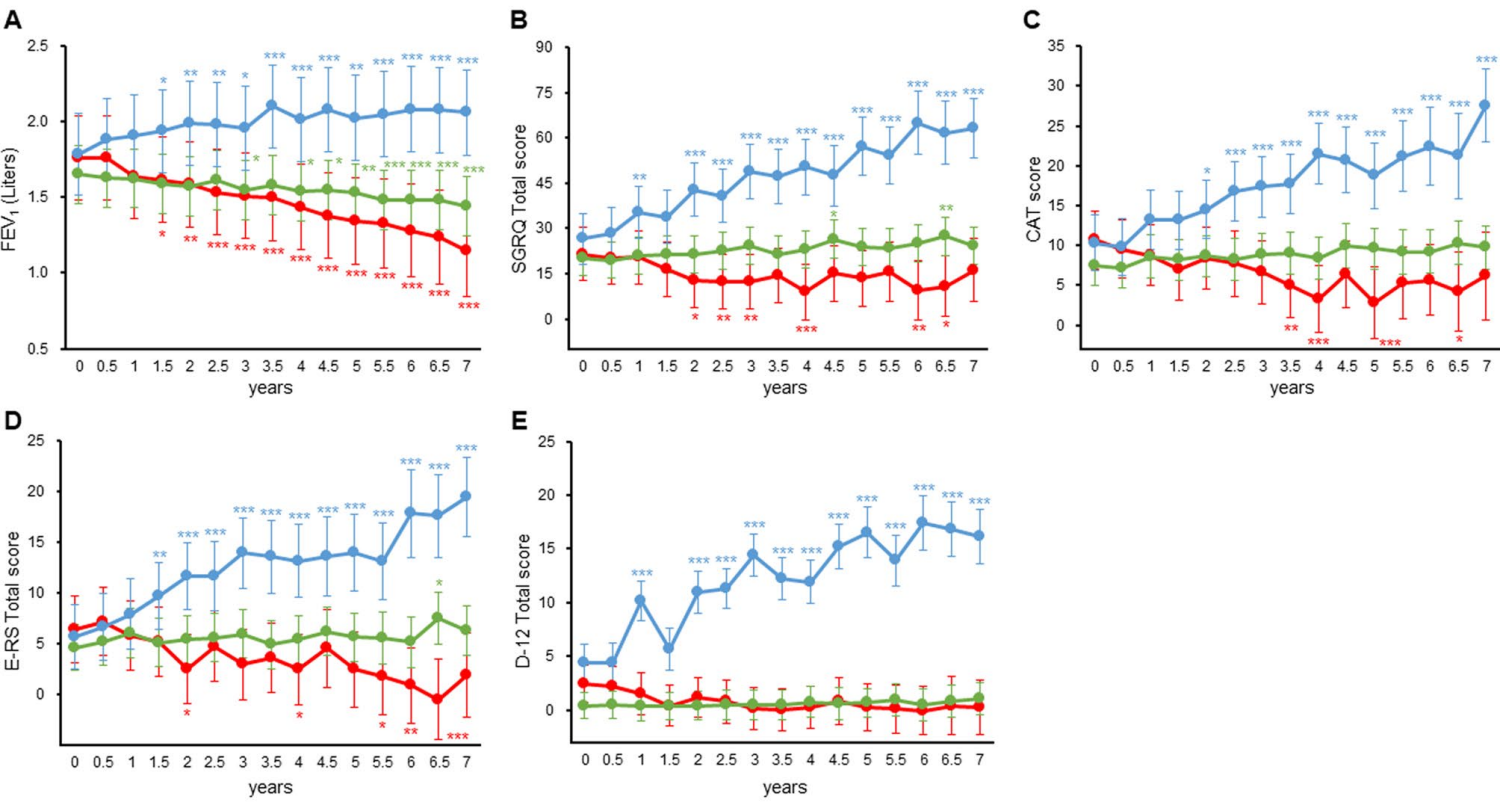
Comparison of the 1st, 2nd and 3rd, and 4th quartiles divided by annual change. The estimated mean and its 95% confidence interval of each indicator by a linear mixed model in the 1st, 2nd and 3rd, and 4th quartiles divided by annual rates of change using linear regression analysis of individual cases for each measurement point every 6 months during a 7-year period. **A**: forced expiratory volume in 1 s (FEV_1_) (Liters), **B**: St. George’s Respiratory Questionnaire (SGRQ) Total score, **C**: COPD Assessment Test (CAT) score, **D**: Evaluating Respiratory Symptoms in COPD (E-RS) Total score, **E**: Dyspnoea-12 (D-12) Total score. ***: *p* < 0.001, **: *p* < 0.01, *: *p* < 0.05 in comparison with baseline. Red line depicts 1st quartile, green shows 2nd and 3rd quartiles and blue indicates 4th quartile

The annual changes in each indicator were analyzed using linear regression to determine the percentile values of the distribution. Patients were divided into three subgroups according to their percentile value: less than the 25th percentile (1st quartile), between the 25th and 75th percentile (2nd and 3rd quartiles), and greater than the 75th percentile (4th quartile). The estimated mean and 95% CI for each subgroup were calculated using a linear mixed model. For each indicator, 17 patients were in the 1st quartile, 35 patients were in the 2nd and 3rd quartiles, and 18 patients were in the 4th quartile. No patients were common in the 1st quartile of FEV_1_ and the 4th quartile of the four PRO measures, while only one was common in the 4th quartile of FEV_1_ and the 1st quartile of the four PRO measures. The estimated means and 95% CI for FEV_1_ for each subgroup over seven years are shown in Fig. 3A and Table S4 in the Additional File. The 1st quartile showed a significant decline from baseline at 1.5 years and continued to decline thereafter. The 4th quartile showed an improvement in FEV_1_ over time, with a significant improvement compared to the baseline at 1.5 years and maintaining that improvement at seven years.

Figure 3B displays plots of the estimated means and 95% CI for each subgroup using a linear mixed model for the SGRQ Total score (Table S5 in the Additional File). The 4th quartile experienced significant worsening from baseline at 1.0 year and continued to deteriorate over time. Conversely, the 1st quartile showed significant improvement compared to baseline at 2.0 years and often continued to improve thereafter. The seven-year trajectory of the CAT score is depicted in Fig. 3C (Table S6 in the Additional File). The 4th quartile exhibited significant worsening from baseline at 2.0 years and continued to deteriorate over time. In contrast, the 1st quartile showed significant improvement from baseline at multiple time points, including 3.5, 4.0, 5.0, and 6.5 years.

In Fig. 3D (Table S7 in the Additional File), the E-RS Total score exhibited significant deterioration from baseline at 1.5 years in the 4th quartile, and this worsening continued over time. However, the 1st quartile showed significant improvement compared to baseline at multiple time points. Figure 3E (Table S8 in the Additional File) illustrates the seven-year progression of the D-12 Total score, with the 4th quartile displaying significant worsening from baseline at 1.0 and 2.0 years, and this continued over time. In contrast, the 1st quartile and the 2nd and 3rd quartiles did not show significant improvement or worsening compared to baseline, indicating that they were relatively stable over the seven-year period.

## Discussion

### Strengths

Over several years, the authors have observed that, as a group, subjects with COPD experience a gradual worsening in health status, respiratory symptoms, and breathlessness. However, a notable discovery from this study is that the rate of decline varies significantly between patients, with some deteriorating rapidly, while others show little to no decline or even a slight improvement over a period of seven years. This study is the first to confirm that the rate of change in indicators of health status, respiratory symptoms, and breathlessness in COPD patients varies greatly. The quartile trajectories based on PRO outcome measures, as well as FEV_1_, showed a progressive decline in one group compared to stability or slight improvement in another group. These findings challenge the notion that COPD always leads to a continuous loss of lung function and clinical decline, suggesting that some patients with COPD can stabilize for long periods with proactive treatment. However, it was also observed that there was a group that followed the traditional course of COPD, with these indicators worsening over time.

Regarding the long-term progression of SGRQ, there are two studies from the 1990s that analyzed data in a similar manner, and both reported reductions in annual decline rates in SGRQ Total scores. One was the ISOLDE study [4, 14, 33], as mentioned earlier, and the other was by Oga et al., which showed a decline rate of 1.87 units/year [34]. Our results showed a slightly smaller decline rate. Oga et al. also reported a decline in SGRQ scores, with the annual change in the Symptoms score not being statistically significant [34], which aligns with our findings.

In the 20th century, it was commonly believed that lung function in COPD deteriorates over time, with various trajectories based on group averages or medians [1, 35]. Vestbo et al. conducted a large study on the rate of FEV_1_ decline over time and were the first to demonstrate that the decline varies considerably among individuals, although they did not show the exact trajectory [36]. Long-term cohort studies to determine the rate of FEV_1_ decline in COPD are challenging due to high dropout rates [37], requiring statistical adjustments to account for dropout and adding complexity to the analysis. In light of this background, the Hokkaido COPD cohort study, a five-year study conducted in Japan, divided participants into quartiles based on the rate of FEV_1_ decline: sustainers (above the 75th percentile), slow decliners (between the 25th and 75th percentile), and rapid decliners (below the 25th percentile), effectively illustrating the five-year trajectory of FEV_1_ [38]. The same analysis used in their study was also applied in the current analysis. The sustainers quartile in the Hokkaido COPD cohort study, characterized by a stable FEV_1_ of around 1.8 L over three years, was compared to the results of the present study. The quartile with the slowest FEV_1_ progression, or 4th quartile, in the present study showed an improvement of over 0.2 L, increasing from 1.8 to over 2.0 L after seven years. Additionally, the SGRQ demonstrated an improvement surpassing the minimal important difference (MID) of 4 in the 1st quartile [39, 40]. The latter shows a more favorable course of COPD.

Vestbo et al. reported that the continuation of smoking is strongly associated with an increased rate of decline in FEV_1_ [36], and the Hokkaido COPD cohort study found that emphysema severity was independently associated with a rapid annual decline in FEV_1_ [38]. Additionally, a significant proportion of subjects who maintained FEV_1_ displayed increased levels of circulating eosinophils [38]. In the current study, all attempts to determine patient characteristics related to the annual rate of change of each indicator over time were negative. However, the weak correlation of baseline diffusion capacity with the annual rates of change in FEV_1_ and SGRQ Total score may be consistent with previous findings that emphysema severity is associated with the rate of deterioration of FEV_1_ [38]. Another important factor that makes the identification of predictors difficult is the lack of correlation between the rate of decline of FEV_1_ and the rate of deterioration of the four PRO measures.

Each PRO measure assesses different aspects of health, and it is important to consider their measurement properties when interpreting the results. We found that in the first quartiles of SGRQ Total, CAT, and E-RS Total scores, there were some instances where the scores improved compared to baseline. However, the D-12 Total score did not show any improvement in the 1st quartile and remained relatively stable throughout the seven years. This could be because the D-12 Total scores were significantly skewed towards the mild side at baseline [28]. The SGRQ and CAT are both tools used to evaluate COPD-specific health status and, while they differ conceptually [41], have similar discriminatory and predictive abilities in clinical settings [25]. From the viewpoint of evaluative property, our study suggests that the SGRQ may be more responsive than the CAT.

Although this study was not initially designed within a specific behavioral theoretical framework, the findings may be interpreted in light of established health psychology models. For instance, the Health Belief Model (HBM) may offer insights into how patients’ perceptions of their disease severity, susceptibility to deterioration, and perceived benefits of treatment adherence or lifestyle changes (e.g., smoking cessation) affect the trajectory of health status and symptoms over time. Similarly, the Theory of Planned Behavior (TPB) might help elucidate how patients’ intentions to maintain or change certain health behaviors—shaped by their attitudes, subjective norms, and perceived control—can influence the long-term management of dyspnea and respiratory symptoms. Future studies could build upon these findings by explicitly incorporating such theoretical frameworks to examine the psychological and behavioral factors contributing to COPD trajectories in more detail.

### Limitations

The current study is limited by its design in several ways. One major limitation is the small number of cases included in the study. A larger sample size could have helped identify which background characteristics are associated with patients whose conditions rapidly progress or remain stable over a seven-year period. However, the study included all eligible patients with stable COPD seen at the hospital during the study period. Another limitation is that acute exacerbations were evaluated but not recorded, preventing analysis of their impact on PRO measures. Exacerbations are known to significantly affect health status [42–45], and it is possible that they played a role in the course of the outcome measures. Conducted at a single center, the findings may not be broadly applicable. The study had more older patients and fewer severe COPD cases compared to previous research. The predominance of male participants limits generalizability to females, reflecting lower COPD prevalence among women in Japan. Improvement in FEV_1_ and PRO measures might be influenced by asthma-COPD overlap (ACO), a definition generally preferred in Japan [46, 47]. Our study excluded patients with a history of asthma but did not address ACO specifically. The exclusion of more ACO-related cases might lead to a more accurate analysis.

## Conclusions

In conclusion, the group as a whole experienced significant deterioration in PRO measures and FEV_1_ over a seven-year period. On average, FEV_1_ declined by 25 milliliters per year, while SGRQ Total and CAT scores worsened by 1.4 and 0.6 units per year, respectively. However, this study highlights the high variability in COPD progression among individuals. While some patients see a worsening in health status, respiratory symptoms, and breathlessness, others remain stable or even improve. This challenges the common belief that COPD leads to continuous deterioration and suggests that proactive treatment can help stabilize the condition for certain patients.

## Electronic supplementary material

Below is the link to the electronic supplementary material.

Supplementary Material 1

## Data Availability

Anonymized participant data will be made available upon reasonable request to the corresponding author.

## Abbreviations

COPD: Chronic obstructive pulmonary disease
PROs: Patient-reported outcomes
SGRQ: St. George’s Respiratory Questionnaire
CAT: COPD Assessment Test
E-RS: Evaluating Respiratory Symptoms in COPD
D-12: Dyspnoea-12
FEV_1_: Forced expiratory volume in one second
ISOLDE: Inhaled Steroids in Obstructive Lung Disease
NCGG: National Center for Geriatrics and Gerontology
FVC: Forced vital capacity
RV: Residual volume
CI: Confidence intervals
GOLD: Global Initiative for Chronic Obstructive Lung Disease
DLco: Diffusion capacity of the lung
MID: Minimal important difference
ACO: Asthma-COPD overlap
