# Supplementary material for: Health status, respiratory symptom and dyspnea trajectories in subjects with chronic obstructive pulmonary disease: a seven-year observation in clinical practice
Source: J Patient Rep Outcomes. 2025 Jul 11;9:88. doi: 10.1186/s41687-025-00923-z (PMC12254445; doi:10.1186/s41687-025-00923-z)
Supplement: Supplementary file 1 — Supplementary Material 1 [file 41687_2025_923_MOESM1_ESM.pdf]

Additional File

Health status, respiratory symptom and dyspnoea trajectories in subjects with chronic obstructive pulmonary disease: a seven-year observation in clinical practice

Table S1. Patient characteristics at baseline and comparison between the groups according to the number of assessments.

	Units	All Patients	No. of Assessments			p-value	
			13 to 15	8 to 12	3 to 7		
		n=70	n=23	n=26	n=21		
Age	years	74.19 ± 0.75	71.3 ± 1.25	75.81 ± 1.16	75.33 ± 1.36	0.060	†
Female sex	number (%)	6 (8.6)	2 (8.7)	3 (11.5)	1 (4.8)	0.868	§
Current smoker	number (%)	16 (22.9)	4 (17.4)	4 (15.4)	8 (38.1)	0.168	§
Cumulative smoking	pack-years	58.27 ± 3.8	51.99 ± 5.4	59.59 ± 8.4	63.51 ± 4.7	0.166	†
Body-mass index (BMI)	kg/m^2	22.22 ± 0.4	22.44 ± 0.5	22.38 ± 0.6	21.79 ± 0.8	0.807	†
FEV_1	Liters	1.71 ± 0.07	1.89 ± 0.13	1.61 ± 0.10	1.65 ± 0.11	0.137	†
FEV_1	%pred	68.7 ± 2.5	72.2 ± 5.1	68.1 ± 3.7	65.6 ± 4.6	0.604	†
TLC	Liters	5.98 ± 0.23	5.91 ± 0.25	6.10 ± 0.55	5.89 ± 0.28	0.494	†
DLco	mL/min/mmHg	12.90 ± 0.65	14.92 ± 1.33	11.81 ± 0.88	12.03 ± 1.07	0.162	†
DLco/V_A	mL/min/mmHg/L	2.87 ± 0.14	3.21 ± 0.23	2.73 ± 0.24	2.67 ± 0.24	0.259	†
PaO_2	torr	79.2 ± 1.1	81.5 ± 1.8	78.9 ± 1.7	77.1 ± 2.0	0.390	†
Blood eosinophil count	%	2.9 ± 0.2	3.0 ± 0.3	2.9 ± 0.5	2.5 ± 0.3	0.563	†
Blood eosinophil count	$cells/mm^3$	182 ± 17	192 ± 27	180 ± 30	172 ± 32	0.715	†
Erythrocyte sedimentation rate	mm/hour	27 ± 2	20 ± 3	31 ± 4	29 ± 4	0.101	†
C-reactive protein[※]	μg/mL	4.6 ± 1.2	7.3 ± 3.3	4.0 ± 1.4	2.5 ± 0.6	0.978	†
Surfactant protein D[※※]	ng/mL	65 ± 8	65 ± 16	65 ± 12	63 ± 12	0.702	†
LABA treatment	number (%)	50 (71.4)	13 (56.5)	20 (76.9)	17 (81.0)	0.181	§
LAMA treatment	number (%)	63 (90.0)	21 (91.3)	23 (88.5)	19 (90.5)	1.000	§
ICS treatment	number (%)	50 (71.4)	13 (56.5)	20 (76.9)	17 (81.0)	0.181	§
SGRQ Total Score	(0-100)	22.2 ± 2.0	19.1 ± 3.2	22.8 ± 3.4	24.9 ± 3.7	0.499	†
SGRQ Symptoms	(0-100)	37.7 ± 2.3	32.9 ± 4.1	38.9 ± 3.9	41.6 ± 3.7	0.287	†
SGRQ Activity	(0-100)	30.2 ± 2.9	27.2 ± 4.6	29.5 ± 4.9	34.4 ± 5.9	0.749	†
SGRQ Impact	(0-100)	13.1 ± 1.7	10.5 ± 3.0	14.1 ± 2.9	14.6 ± 3.1	0.409	†
CAT Score	(0-40)	9.0 ± 0.8	6.8 ± 1.0	9.3 ± 1.5	11.1 ± 1.6	0.174	†
E-RS Total Score	(0-40)	5.4 ± 0.7	3.9 ± 1.0	5.5 ± 1.2	6.8 ± 1.5	0.306	†
RS-Breathlessness	(0-17)	2.5 ± 0.4	1.8 ± 0.7	2.7 ± 0.7	3.0 ± 0.8	0.446	†
RS-Cough & Sputum	(0-11)	1.9 ± 0.2	1.4 ± 0.3	1.9 ± 0.4	2.5 ± 0.4	0.135	†
RS-Chest Symptoms	(0-12)	1.0 ± 0.2	0.7 ± 0.3	0.9 ± 0.3	1.3 ± 0.4	0.612	†
D-12 Total Score	(0-36)	1.9 ± 0.4	1.5 ± 0.8	2.2 ± 0.7	2.0 ± 0.6	0.280	†
D-12 Physical Score	(0-21)	1.6 ± 0.3	1.1 ± 0.5	1.9 ± 0.5	1.8 ± 0.5	0.257	†
D-12 Affective Score	(0-15)	0.3 ± 0.1	0.4 ± 0.3	0.4 ± 0.2	0.2 ± 0.2	0.985	†

mean±SE, †Kruskal-Wallis test, §Fisher's exact test, [※] (from left to right) n=68, 22, 25, 21, [※※] (from left to right) n=67, 22, 26, 19, LABA, long-acting beta-agonists; LAMA, Tiotropium; ICS, inhaled glucocorticoids; SGRQ, the St. George's Respiratory Questionnaire; CAT, the COPD Assessment Test; E-RS, the Evaluating Respiratory Symptoms in COPD; D-12, Dyspnoea-12. The numbers in parentheses denote possible score range.

Table S2. Transits in measured values over seven years, as calculated using a linear mixed model.
The estimated mean and its 95% confidence interval of the FEV_1, SGRQ Total, CAT, E-RS Total and D-12 Total scores by a linear mixed model for each measurement point every 6 months during a 7-year period.

years	FEV_1 (L)	SGRQ Total score	CAT score	E-RS Total score	D-12 Total score
0.0	1.71 (1.57-1.85)	22.2 (17.4-27.0)	9.0 (7.1-11.0)	5.4 (3.6-7.1)	1.9 (0.5-3.3)
0.5	1.72 (1.58-1.86)	22.5 (17.7-27.4)	8.6 (6.6-10.6)	6.1 (4.4-7.9)	2.1 (0.7-3.5)
1.0	1.69 (1.55-1.83)	24.5 (19.6-29.5)	9.8 (7.8-11.9)	6.5 (4.7-8.4)	3.3 (1.8-4.8)
1.5	1.68 (1.54-1.82)	23.7 (18.8-28.6)	9.2 (7.2-11.2)	6.4 (4.6-8.2)	1.9 (0.4-3.3)
2.0	1.68 (1.54-1.82)	24.6 (19.7-29.6)	10.0 (8.0-12.0)	6.3 (4.5-8.1)	3.4 (1.9-4.8)
2.5	1.68 (1.54-1.82)	24.6 (19.6-29.5)	10.4 (8.3-12.4)	6.8 (5.0-8.6)	3.5 (1.9-5.0)
3.0	1.63 (1.49-1.77)	26.9 (21.9-31.9) *	10.5 (8.5-12.6)	6.9 (5.1-8.8)	3.8 (2.2-5.3) *
3.5	1.67 (1.53-1.82)	25.5 (20.6-30.5)	10.4 (8.3-12.5)	6.5 (4.6-8.3)	3.3 (1.8-4.8)
4.0	1.62 (1.48-1.76) **	25.6 (20.6-30.6)	10.7 (8.6-12.8)	6.4 (4.6-8.3)	3.4 (1.9-4.9)
4.5	1.63 (1.48-1.77)	28.3 (23.2-33.5) **	11.9 (9.7-14.1) *	7.4 (5.5-9.3)	4.3 (2.7-5.9) **
5.0	1.59 (1.45-1.73) ***	28.1 (23.0-33.2) **	10.7 (8.6-12.9)	6.7 (4.8-8.6)	4.1 (2.5-5.7) *
5.5	1.58 (1.44-1.73) **	28.5 (23.2-33.7) **	11.1 (8.9-13.4)	6.4 (4.4-8.4)	3.9 (2.2-5.5)
6.0	1.59 (1.45-1.74) **	29.0 (23.8-34.3) **	11.2 (8.9-13.4)	6.4 (4.4-8.3)	4.1 (2.5-5.8) *
6.5	1.59 (1.44-1.73) **	30.7 (25.4-36.1) ***	11.6 (9.3-14.0)	7.9 (5.9-9.9) *	4.5 (2.8-6.2) **
7.0	1.54 (1.39-1.68) ***	30.4 (25.2-35.7) ***	12.7 (10.4-14.9) **	8.2 (6.2-10.2) **	4.4 (2.7-6.0) **

***: $p<0.001$, **: $p<0.01$, *: $p<0.05$ in comparison with baseline, SGRQ, the St. George's Respiratory Questionnaire; CAT, the COPD Assessment Test; E-RS, Evaluating Respiratory Symptoms in COPD; D-12, Dyspnoea-12.

Table S3. Patient characteristics at baseline on annual rate of change.
Effects of patient characteristics at baseline on annual rate of change in FEV_1 as well as the SGRQ Total, CAT, E-RS Total and D-12 Total scores.

	FEV_1 (mL)		SGRQ Total Score		CAT Score		E-RS Total Score		D-12 Total Score	
	Effect on Annual Rate of Change (ml/yr)	p-value	Effect on Annual Rate of Change (/yr)	p-value	Effect on Annual Rate of Change (/yr)	p-value	Effect on Annual Rate of Change (/yr)	p-value	Effect on Annual Rate of Change (/yr)	p-value
Age (per yr)	-1.824	0.196	0.032	0.670	-0.031	0.526	-0.026	0.583	0.001	0.976
Female sex	2.659	0.933	-1.728	0.297	-1.056	0.326	-1.023	0.337	-0.745	0.347
Current smoker (yes vs. no)	-4.905	0.816	-0.491	0.658	-0.385	0.593	-0.298	0.676	0.639	0.225
Smoking history (per pack-yr)	0.156	0.575	0.010	0.479	0.003	0.748	-0.003	0.775	-0.003	0.709
LABA treatment (yes vs. no)	-6.709	0.732	0.894	0.384	-0.331	0.621	-0.352	0.595	0.136	0.782
LAMA treatment (yes vs. no)	-36.964	0.208	-0.715	0.645	1.006	0.316	0.452	0.650	0.298	0.687
ICS treatment (yes vs. no)	-6.709	0.732	0.894	0.384	-0.331	0.621	-0.352	0.595	0.136	0.782

LABA, long-acting beta-agonists; LAMA, Tiotropium; ICS, inhaled glucocorticoids; SGRQ, the St. George’s Respiratory Questionnaire; CAT, the COPD Assessment Test; E-RS, the Evaluating Respiratory Symptoms in COPD; D-12, Dyspnoea-12.

Table S4. Comparison of the quartiles of FEV_1 divided by annual change.
The estimated mean and its 95% confidence interval of the FEV_1 in the 1st, 2nd and 3rd, and 4th quartiles by a linear mixed model for each measurement point every 6 months during a 7-year period.

years	1st quartile	2nd and 3rd quartiles	4th quartile
0.0	1.76 (1.48-2.04)	1.65 (1.46-1.85)	1.79 (1.52-2.06)
0.5	1.76 (1.48-2.04)	1.63 (1.43-1.82)	1.89 (1.62-2.16)
1.0	1.64 (1.36-1.92)	1.63 (1.43-1.82)	1.91 (1.64-2.18)
1.5	1.62 (1.34-1.90) *	1.59 (1.39-1.78)	1.94 (1.67-2.21) *
2.0	1.59 (1.30-1.87) **	1.57 (1.38-1.77)	1.99 (1.71-2.27) **
2.5	1.54 (1.26-1.82) ***	1.61 (1.42-1.81)	1.98 (1.70-2.26) **
3.0	1.51 (1.23-1.79) ***	1.55 (1.35-1.74) *	1.96 (1.68-2.24) *
3.5	1.50 (1.22-1.78) ***	1.58 (1.38-1.78)	2.10 (1.83-2.38) ***
4.0	1.44 (1.16-1.72) ***	1.54 (1.35-1.74) *	2.02 (1.74-2.29) ***
4.5	1.38 (1.09-1.67) ***	1.55 (1.35-1.74) *	2.08 (1.80-2.36) ***
5.0	1.34 (1.06-1.63) ***	1.53 (1.33-1.72) **	2.03 (1.74-2.31) **
5.5	1.33 (1.03-1.62) ***	1.48 (1.29-1.68) ***	2.05 (1.77-2.33) ***
6.0	1.28 (0.97-1.59) ***	1.48 (1.29-1.68) ***	2.08 (1.80-2.37) ***
6.5	1.24 (0.93-1.55) ***	1.48 (1.29-1.68) ***	2.08 (1.79-2.36) ***
7.0	1.14 (0.84-1.45) ***	1.44 (1.25-1.64) ***	2.06 (1.78-2.35) ***

***: $p<0.001$, **: $p<0.01$, *: $p<0.05$ in comparison with baseline.

Table S5. Comparison of the quartiles of SGRQ Total score divided by annual change. The estimated mean and its 95% confidence interval of the SGRQ Total scores in the 1st, 2nd and 3rd, and 4th quartiles by a linear mixed model for each measurement point every 6 months during a 7-year period.

years	1st quartile	2nd and 3rd quartiles	4th quartile
0.0	21.6 (12.9-30.2)	20.3 (14.2-26.3)	26.6 (18.2-35.0)
0.5	20.2 (11.5-28.9)	19.5 (13.4-25.5)	28.5 (19.9-37.1)
1.0	20.4 (11.6-29.3)	20.9 (14.7-27.2)	35.3 (26.7-43.9) **
1.5	16.4 (7.5-25.3)	21.3 (15.1-27.4)	33.8 (25.1-42.5)
2.0	12.7 (3.7-21.6) *	21.4 (15.3-27.5)	42.9 (34.1-51.6) ***
2.5	12.4 (3.4-21.3) **	22.5 (16.4-28.7)	40.7 (31.9-49.6) ***
3.0	12.2 (3.3-21.2) **	24.4 (18.2-30.6)	48.9 (39.9-57.8) ***
3.5	14.6 (5.5-23.6)	21.4 (15.2-27.6)	47.3 (38.4-56.3) ***
4.0	8.9 (-0.3-18.0) ***	22.8 (16.7-29.0)	50.3 (41.1-59.6) ***
4.5	15.1 (6.0-24.2)	26.4 (20.1-32.7) *	47.5 (37.5-57.5) ***
5.0	13.5 (4.3-22.8)	24.0 (17.8-30.1)	57.2 (47.6-66.9) ***
5.5	15.5 (5.6-25.3)	23.6 (17.2-30.0)	54.3 (44.8-63.7) ***
6.0	9.6 (-0.2-19.4) **	25.0 (18.8-31.3)	65.1 (54.6-75.5) ***
6.5	10.9 (0.8-21.0) *	27.4 (21.0-33.8) **	61.6 (51.2-72.1) ***
7.0	16.2 (5.6-26.8)	24.3 (18.0-30.6)	63.2 (53.6-72.9) ***

***: $p<0.001$, **: $p<0.01$, *: $p<0.05$ in comparison with baseline.

Table S6. Comparison of the quartiles of CAT score divided by annual change. The estimated mean and its 95% confidence interval of the CAT scores in the 1st, 2nd and 3rd, and 4th quartiles by a linear mixed model for each measurement point every 6 months during a 7-year period.

years	1st quartile	2nd and 3rd quartiles	4th quartile
0.0	10.7 (7.1-14.3)	7.5 (5.0-10.1)	10.3 (6.8-13.9)
0.5	9.6 (5.9-13.2)	7.2 (4.7-9.8)	9.8 (6.2-13.4)
1.0	8.8 (5.0-12.5)	8.6 (6.0-11.3)	13.2 (9.5-17.0)
1.5	7.0 (3.2-10.8)	8.2 (5.6-10.8)	13.2 (9.6-16.8)
2.0	8.4 (4.5-12.3)	8.7 (6.1-11.3)	14.5 (10.8-18.3) *
2.5	7.7 (3.6-11.9)	8.3 (5.7-10.9)	16.7 (13.0-20.4) ***
3.0	6.7 (2.7-10.6)	8.8 (6.2-11.5)	17.4 (13.6-21.1) ***
3.5	5.0 (1.0-8.9) **	9.0 (6.3-11.7)	17.8 (14.0-21.5) ***
4.0	3.2 (-0.9-7.4) ***	8.4 (5.8-11.0)	21.5 (17.7-25.3) ***
4.5	6.4 (2.2-10.5)	9.9 (7.2-12.7)	20.7 (16.6-24.8) ***
5.0	2.8 (-1.6-7.3) ***	9.6 (6.9-12.2)	18.8 (14.7-22.9) ***
5.5	5.3 (0.9-9.8)	9.2 (6.4-11.9)	21.2 (16.8-25.6) ***
6.0	5.7 (1.2-10.1)	9.2 (6.5-11.9)	22.4 (17.5-27.4) ***
6.5	4.2 (-0.8-9.2) *	10.3 (7.6-13.0)	21.2 (15.8-26.6) ***
7.0	6.2 (0.7-11.6)	9.7 (7.1-12.4)	27.6 (23.0-32.2) ***

***: $p<0.001$, **: $p<0.01$, *: $p<0.05$ in comparison with baseline.

Table S7. Comparison of the quartiles of E-RS Total score divided by annual change. The estimated mean and its 95% confidence interval of the E-RS Total scores in the 1st, 2nd and 3rd, and 4th quartiles by a linear mixed model for each measurement point every 6 months during a 7-year period.

years	1st quartile	2nd and 3rd quartiles	4th quartile
0.0	6.5 (3.2-9.8)	4.7 (2.4-6.9)	5.7 (2.5-8.9)
0.5	7.2 (3.9-10.5)	5.2 (2.9-7.5)	6.7 (3.4-9.9)
1.0	5.9 (2.5-9.3)	6.1 (3.7-8.5)	7.9 (4.5-11.4)
1.5	5.2 (1.8-8.7)	5.1 (2.8-7.5)	9.7 (6.4-13.0) **
2.0	2.6 (-0.9-6.0) *	5.4 (3.0-7.8)	11.7 (8.3-15.0) ***
2.5	4.7 (1.3-8.2)	5.6 (3.2-8.0)	11.7 (8.3-15.1) ***
3.0	3.0 (-0.5-6.4)	6.0 (3.6-8.4)	14.0 (10.4-17.5) ***
3.5	3.6 (0.2-7.1)	4.9 (2.5-7.3)	13.6 (10.0-17.2) ***
4.0	2.5 (-1.0-6.0) *	5.4 (3.0-7.8)	13.2 (9.6-16.8) ***
4.5	4.6 (0.7-8.4)	6.2 (3.8-8.6)	13.6 (9.7-17.5) ***
5.0	2.5 (-1.2-6.2)	5.7 (3.3-8.0)	14.0 (10.2-17.7) ***
5.5	1.8 (-2.0-5.6) *	5.6 (3.0-8.1)	13.2 (9.4-16.9) ***
6.0	0.9 (-2.8-4.6) **	5.2 (2.7-7.6)	17.9 (13.6-22.2) ***
6.5	-0.5 (-4.4-3.5) ***	7.5 (5.0-10.1) *	17.7 (13.6-21.7) ***
7.0	1.9 (-2.2-6.1)	6.3 (3.8-8.8)	19.5 (15.6-23.4) ***

***: p<0.001, **: p<0.01, *: p<0.05 in comparison with baseline.

Table S8. Comparison of the quartiles of D-12 Total score divided by annual change. The estimated mean and its 95% confidence interval of the D-12 Total scores in the 1st, 2nd and 3rd, and 4th quartiles by a linear mixed model for each measurement point every 6 months during a 7-year period.

years	1st quartile	2nd and 3rd quartiles	4th quartile
0.0	2.5 (0.7-4.2)	0.4 (-0.8-1.6)	4.4 (2.7-6.1)
0.5	2.2 (0.5-4.0)	0.5 (-0.8-1.7)	4.4 (2.6-6.2)
1.0	1.5 (-0.4-3.5)	0.4 (-1.0-1.8)	10.1 (8.3-12.0) ***
1.5	0.4 (-1.4-2.3)	0.4 (-0.9-1.7)	5.6 (3.7-7.6)
2.0	1.1 (-0.7-3.0)	0.4 (-0.9-1.7)	11.0 (9.0-13.0) ***
2.5	0.8 (-1.2-2.8)	0.5 (-0.9-1.8)	11.3 (9.4-13.2) ***
3.0	0.1 (-1.9-2.1)	0.5 (-0.9-1.8)	14.4 (12.4-16.4) ***
3.5	0.0 (-2.0-2.0)	0.5 (-0.9-1.9)	12.2 (10.3-14.2) ***
4.0	0.2 (-1.7-2.2)	0.8 (-0.6-2.2)	11.9 (9.9-13.9) ***
4.5	0.8 (-1.3-3.0)	0.6 (-0.9-2.1)	15.2 (13.1-17.2) ***
5.0	0.2 (-2.0-2.4)	0.7 (-0.7-2.0)	16.5 (14.2-18.9) ***
5.5	0.1 (-2.2-2.3)	0.9 (-0.6-2.4)	13.9 (11.6-16.3) ***
6.0	0.0 (-2.3-2.2)	0.5 (-1.0-2.0)	17.4 (14.9-20.0) ***
6.5	0.4 (-2.3-3.1)	0.8 (-0.7-2.4)	16.8 (14.3-19.4) ***
7.0	0.3 (-2.3-2.8)	1.0 (-0.4-2.5)	16.1 (13.6-18.7) ***

***: $p<0.001$ in comparison with baseline.
